# Isolation of Bacteria from Freeze-Dried Samples and the Functional Characterization of Species-Specific Lactic Acid Bacteria with a Comparison of Wild and Captive Proboscis Monkeys

**DOI:** 10.3390/microorganisms11061458

**Published:** 2023-05-31

**Authors:** Nami Suzuki-Hashido, Sayaka Tsuchida, Akinori Azumano, Benoit Goossens, Diana A. Ramirez Saldivar, Danica J. Stark, Augustine Tuuga, Kazunari Ushida, Ikki Matsuda

**Affiliations:** 1 College of Bioscience and Biotechnology, Chubu University, Kasugai 487-8501, Aichi, Japan; 2 Japan Society for the Promotion of Science, Chiyoda-ku 102-0083, Tokyo, Japan; 3 Yokohama Zoo Zoorasia, Yokohama 241-0001, Kanagawa, Japan; 4 Sabah Wildlife Department, Wisma Muis, Kota Kinabalu 88100, Malaysia; 5 Organisms and Environment Division, Cardiff School of Biosciences, Cardiff University, Cardiff CF10 3AX, UK; 6 Danau Girang Field Centre, c/o Sabah Wildlife Department, Wisma Muis, Kota Kinabalu 88100, Malaysia; 7 Wildlife Research Center, Kyoto University, Sakyo-ku 606-8203, Kyoto, Japan; 8 Academy of Emerging Sciences, Chubu University, Kasugai 487-8501, Aichi, Japan; 9 Chubu Institute for Advanced Studies, Chubu University, Kasugai 487-8501, Aichi, Japan; 10 Institute for Tropical Biology and Conservation, University Malaysia Sabah, Kota Kinabalu 88100, Malaysia

**Keywords:** *Lactobacillus nasalidis*, proboscis monkey, phenotypic characteristics, foregut contents, NaCl tolerance

## Abstract

Previously, we isolated a novel lactic acid bacteria species (*Lactobacillus nasalidis*) from the fresh forestomach contents of a captive proboscis monkey (*Nasalis larvatus*) in a Japanese zoo. In this study, we isolated two strains of *L. nasalidis* from the freeze-dried forestomach contents of a wild proboscis monkey inhabiting a riverine forest in Malaysia. The samples had been stored for more than six years. Phenotypic analysis showed that strains isolated from the wild individual had more diverse sugar utilization and lower salt tolerance than strains previously isolated from the captive counterpart. These phenotypic differences are most likely induced by feeding conditions; wild individuals consume a wide variety of natural food, unlike their zoo-raised counterparts that consume formula feed with sodium sufficiency. Since 16s rRNA sequences of *L. nasalidis* were detected in the previously created 16S rRNA libraries of wild, provisioned, and captive proboscis monkeys in Malaysia and Japan, *L. nasalidis* may be an essential bacterium of the foregut microbial community of the proboscis monkey. The currently established method for the isolation of gut bacteria from freeze-dried samples under storage will be applicable to many already-stored precious samples.

## 1. Introduction

The gastrointestinal tract (GIT) microbiota plays important roles in host health in terms of food digestion, nutrient metabolism, immune system development, pathogen resistance, and other important aspects of the host’s health [1]. Lactic acid bacteria (LAB), a group of microaerophilic and Gram-positive organisms, which ferment hexose sugars to produce primarily lactic acid [2], are well studied in GIT microbiota. This is because of their health benefits to host animals, such as immune modulation, the alleviation of allergies, and the exclusion of pathogens [3,4,5]. In addition to the family *Lactobacillaceae*, the genus *Bifidobacterium* is often regarded as lactate-producing beneficial bacteria in the gut of mammals, particularly in humans [6]. Among the LAB, the genus *Lactobacillus* is a big bacterial group that is comprised of 261 species as of March 2020. Recently, the genus *Lactobacillus* has been reclassified into 25 genera [7]. Lactic acid bacteria (LAB) have adapted to vertebrate and invertebrate hosts as autochthonous species in the GIT [8], and there are host-specific LAB in the GIT.

It is well known that the feeding habits of the host—herbivory, omnivory, or carnivory—shape the composition of GIT-LAB [9,10,11]. In addition to the difference in species-level composition of LAB in the GIT, individual strains of the same LAB species isolated from the same host species that lived in different environments, for example, in the wild and under captivity, showed a significant difference in physiological characteristics in terms of sugar utilization and salt tolerance [12]. The feeding condition therefore clearly selects for the strains that have adapted better to the GIT environment. Consequently, the functional analysis of LAB has a considerable potential for the better understanding of the ecophysiology of the host. However, such strain-level comparative studies are based on the isolation and culturing of GIT bacteria in wild animals. These studies remain very limited due to the difficulty of laboratory-level studies in the field [13]. In this study, we tried to isolate GIT LAB from the long-term preserved foregut contents of wild proboscis monkeys, as a good alternative to study the functionality of GIT bacteria in the wild free-ranging animals at the strain level. Since we have already discovered a novel species of the genus *Lactobacillus* (*Lactobacillus nasalidis*), isolated from the fresh forestomach contents of a proboscis monkey (*Nasalis larvatus*) that was held captive in a Japanese zoo [14], we targeted the exploration of this particular LAB in the wild proboscis monkey.

Proboscis monkeys, a member of the subfamily Colobinae, are endemic to Borneo, the largest island in Asia, and inhabit mangroves, peat swamps, and riverine forests [15]. As in the case of other colobine species, proboscis monkeys are primarily folivorous and have a multi-chambered stomach [16]. Since the pH of the forestomach content is regulated to a weakly acidic environment at approximately 5.5 to 7 [17,18], colobine monkeys harbor a wide variety of gut bacteria in their forestomach [19,20] and use these symbiotic bacteria to ferment undigestible plant fiber [21]. Among the colobine monkeys, the proboscis monkeys are unique colobines with ruminating behaviors [22]. From our previous high-throughput 16S rRNA sequencing analysis of the forestomach contents, wild proboscis monkeys inhabiting environments with complex vegetation showed a higher diverse microbiome than the captive individuals that are allowed to access a very limited number of food items [19]. In addition to this culture-independent approach, a comparison of physiological properties between strains of *L. nasalidis* isolated from wild, semi-wild, and captive proboscis monkeys can reveal the adaptation of GIT LAB to the feeding environment, which we made in a previous study with gorillas [12]. We therefore carried out detailed functional analyses of *L. nasalidis* wild isolates by comparing their ability to ferment various sugars, including plant secondary metabolic compounds, with five previously isolated *L. nasalidis* strains, including type strain JCM 33769^T^ [14].

## 2. Materials and Methods

### 2.1. Isolation of Bacteria from Freeze-Dried Samples of Wild, Semi-Wild, or Captive Proboscis Monkeys

We collected forestomach contents of proboscis monkeys living in different environmental conditions, which is to say, wild, semi-wild, and captivity, in Sabah, Malaysian Borneo, between April 2013 and June 2014 (see Hayakawa et al. [19] for details). The samples were immediately frozen at −80 °C and shipped to the laboratory. All samples were later freeze-dried in July 2015. In December 2019, we attempted to isolate *L. nasalidis* from the freeze-dried samples that had been stored at −20 °C for more than four years. A total of eight samples collected from five adult males were analyzed: four from two wild individuals in a riverine forest (part of the Kinabatangan River), one from a wild individual in a mangrove forest (part of the Kinabatangan River), one from a provisioned semi-wild individual in a mangrove forest of the Labuk Bay Proboscis Monkey Sanctuary, and two from one captive individual in the Lok Kawi Wildlife Park. All the freeze-dried samples were moderately diluted with cooled SOC medium and then inoculated into anaerobic-modified GAM broth (Nissui Pharmaceutical Co., Ltd., Tokyo, Japan). After overnight incubation at 37 °C, while maintaining anaerobic conditions using the Anaero pouches (Mitsubishi Gas Chemical Company, Inc., Tokyo, Japan), these samples were inoculated onto MRS agar plates (BD Difco, Fisher Scientific, Ottawa, ON, Canada). Colonies that grew on the plates were then purified in the same medium with several transfers to a fresh medium. The isolates were grown on MRS agar plates and used for DNA extraction.

### 2.2. Phylogenetic Analysis of the 16S rRNA Gene

After bead disruption, DNA was extracted using a commercial kit (QuickGene-Mini80, Kurabo Industries Ltd., Osaka, Japan). A nearly complete length of 16S rRNA gene sequences of the isolates was determined as previously described [23]. These amplicons were sequenced at Hokkaido System Science Co., Ltd. (Sapporo, Japan) using the dye-terminator method. To reconstruct a phylogenetic tree, sequences close to that of *L. nasalidis* were retrieved from public databases. Multiple alignments of the sequences were carried out using the ClustalW program [24]. Phylogenetic trees for a nearly complete length of 16S rRNA genes were constructed using the neighbor-joining method [25] with Kimura’s two-parameter model [26], and the tree topologies were evaluated using a bootstrap analysis with 1000 replicates. These analyses were conducted in MEGA X [27].

### 2.3. Molecular Confirmation of the Presence of L. nasalidis in the Previous 16S Metagenomic DNA Library

The presence of *L. nasalidis* in the freeze-dried samples was confirmed by a molecular approach. We obtained the V1–V2 region (approximately 340 bp) of the 16S rRNA sequences of *Lactobacillaceae*, including *L. nasalidis*, from the 16S metagenomic DNA library constructed in our previous study [19]. We then constructed a phylogenetic tree of the V1–V2 region to define the presence of *L. nasalidis* in the foregut samples.

### 2.4. Phenotypic Characterization of Isolated Bacteria

Physiological and biochemical characteristics were evaluated using the API 50 CHL and API ZYM systems (bioMerieux, Marcy-l’Étoile, France) following the manufacturer’s instructions. The enzyme reactions were divided into six grades, as follows: negative (grade 0), weakly positive (grades 1–2), and positive reactions (grades 3–5). A growth-range test was conducted in MRS broth (BD Difco) at 15 °C and 45 °C for seven days under anaerobic conditions. Tolerance to NaCl was examined according to the parameters of Tsuchida et al. [12] in MRS broth containing 1, 2, 3, 4, 5, 6, 8, and 10% (*w*/*v*) NaCl after incubation for seven days at 37 °C. These tests were repeated two times. Finally, to understand the intraspecific variation in the phenotypic characteristics of bacteria, comparisons were made between the strains from samples collected in the present study and five previously isolated strains, including the typed strain JCM 33769^T^ (=YZ01^T^) from the forestomach contents of a captive proboscis monkey in Japan [14].

## 3. Results and Discussions

### 3.1. Isolation of L. nasalidis from the Freeze-Dried Samples of a Wild Proboscis Monkey

**Figure 1:**
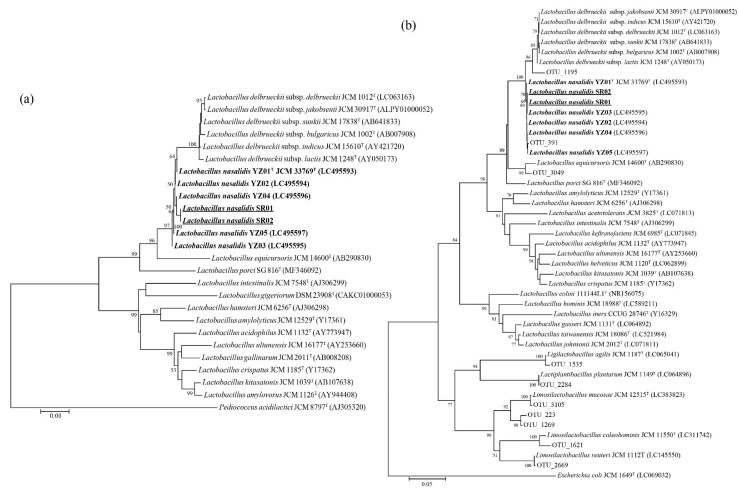
Phylogenetic tree of *Lactobacillus nasalidis* isolated from wild and captive individuals and closely related type strains: (**a**) Phylogenetic tree based on nearly complete length (approx. 1500 bp) of 16S rRNA gene sequence. (**b**) Phylogenetic tree of *L. nasalidis*, related species, and 10 OTU sequences of *Lactobacillaceae* in the DNA library in Hayakawa et al. [19] based on V1–V2 region (approx. 340 bp) of 16S rRNA gene sequences. These trees were constructed by the neighbor-joining method with Kimura’s two-parameter model. Bootstrap values (>50%) based on 1000 replicates are shown at branch nodes. Bold and underline indicate *Lactobacillus nasalidis* and strains isolated in this study, respectively.

Of the eight previously frozen forestomach samples collected from five individuals, several GIT bacteria were successfully cultured—two from a wild individual in the riverine forest, one from a semi-wild individual in a mangrove forest, and two from a captive individual in a Malaysian zoo. However, attempts failed for three samples—two from a wild individual in the riverine forest and one from a wild individual in a mangrove forest. Two strains of *L. nasalidis* were isolated from a single sample collected from a wild individual in the riverine forest and assigned as strains SR01 and SR02. SR01 and SR02 had identical 16S rRNA sequences. The phylogenetic tree of the 16S rRNA gene sequences of *L. nasalidis* and related species (Figure 1a) showed that strains SR01 and SR02 had the most similarities to strain YZ01^T^ (99.93%), which is the type strain of *L. nasalidis* previously detected in a captive proboscis monkey in Japan [14]. However, *L. nasalidis* was not isolated from the other four samples. Other *Lactobacillaceae* species, i.e., *Limosilactobacillus reuteri*, *Limosilactobacillus colehominis*, and *Limosilactobacillus mucosae*, were isolated from a captive individual in a Malaysian zoo.

Here, we successfully isolated *L. nasalidis* from the freeze-dried forestomach contents of a wild individual that had been stored over a six-year period, which is to say, approximately two years of freezing at −80 °C and a subsequent four-year period of the freeze-dried samples stored at −20 °C. In a past study, there were no ill effects of preservation on aerobes, anaerobes, and bifidobacteria that had been archived under aerobic conditions at −80 °C for a week [28]. Freeze-dried preparations of LAB are commonly used industrially because of their successful long-term preservation and handling convenience [29]. However, bacterial isolation from frozen and subsequently freeze-dried samples such as feces and GIT contents has not been widely reported, so far. Our results may offer another approach for the bacteriology of wild animals in remote areas, where it is difficult to culture and isolate them immediately [13].

In this study, the samples from which we could successfully isolate *L. nasalidis* were originally collected for DNA analysis, not for culture analysis. Freeze-dried samples are often used for DNA analysis, such as high-throughput 16S rRNA sequencing analysis [30], because freeze-drying stabilizes samples and reduces their weight, reducing the risk and cost of shipping samples over long distances [31]. As shown by this study, the currently established method for the isolation of bacteria from freeze-dried samples will be applicable to many samples that have already been collected and stored for DNA analysis. Our method has the potential to enrich knowledge about GIT bacteria in wild animals by using stored freeze-dried samples that would be difficult to collect again.

In addition to *L. nasalidis*, we successfully isolated other Gram-positive bacteria from four samples such as *Clostridium paraputrificum*, *Enterococcus casseliflavus*, *Paraclostridium bifermentans*, *Paraclostridium benzoelyticum*, *Streptococcus gallolyticus*, and *Streptococcus lutetiensis* and previously unknown *Lactobacillaceae* species. This isolation record suggests that the present isolation method may be limited for Gram-positive aerotolerant bacteria. In fact, it has been reported that the survival rate of Gram-positive bacteria after freeze-drying tends to be higher than that of Gram-negative bacteria at the same point in time, due to the structure of the cell surface [32]. Interestingly, we have isolated a Gram-negative bacterium, a previously unknown *Prevotellaceae* strain, from the same sample from which *L. nasalidis* was isolated. This sample was collected from a wild individual in the riverine forest. Although the presently established method is mostly applicable to the isolation of Gram-positive aerotolerant species, in some cases, this method can rescue highly Gram-negative anaerobic species. In fact, one of the authors of this manuscript successfully recovered *Bacteroides xylanosolvens* and *Bacteroides ovatus* from a freeze-dried five-year stored fecal sample of a Japanese macaque (*Macaca fuscata*). As yet, however, no interpretation is currently available as to the reason behind the detection of Gram-negative, highly anaerobic species in freeze-dried fecal samples. Such an explanation is still needed to further improve the long-term preservation and rescue methodology for less-culturable bacterial species.

### 3.2. Comparison of L. nasalidis Proportion between Different Habitat Environments in the 16S DNA Library

When reanalyzed, the previously constructed 16S metagenomic DNA library of Hayakawa et al. [19] with the V1–V2 regions of 16S rRNA genes, 10 operational taxonomic units (OTUs) sequences of *Lactobacillaceae* were obtained from the wild (in a riverine forest), semi-wild (in a mangrove forest), and captive individuals (Table 1). Among them, OTU 391 showed high similarity (only two substitutions) to SR01 and SR02, in addition to YZ01^T^ (Figure 1b). The operational taxonomic unit (OTU) 391 shared eight reads, 277 reads, and 148 reads in the libraries of wild individuals, semi-wild provisioned individuals, and captive individuals of the Malaysian samples, respectively (Table 1). The OTU 391 was the only OTU assigned as *Lactobacillaceae* in the wild individuals (100%) in the riverine forest; the proportion was still high (89.9%) in the semi-wild individuals. However, the proportion of OTU 391 declined tremendously in the captive individuals (12.7%), and the value was significantly lower than both for wild riverine forest and semi-wild mangrove forest (*p* < 0.00001, Chi-square test). No reads of *Lactobacillaceae* were obtained for the wild individual inhabiting a mangrove forest.

Although the proportion of *L. nasalidis* to the total *Lactobacillaceae* determined through the sequence library data varied across habitats, the fact that *L. nasalidis* was not only detected in the individuals with natural, provisioned, and captive conditions in Malaysia but also in those kept in a long-term captive condition in Japan, suggests that *L. nasalidis* may be an essential keystone or core bacterium of the foregut microbial community of the proboscis monkey. The 16S rRNA sequence of OTU391, with the highest similarity to *L. nasalidis*, was detected in the wild, semi-wild, and zoo individuals, with one exception of a wild individual in a mangrove forest (Table 1). Wild proboscis monkeys living in mangrove forests presented in this study consumed only seven plant species, including seven genera and seven families and they subsisted primarily on leaves and unripe fruits of a single plant species, *Sonneratia caseolaris* [33]. On the other hand, the provisioned semi-wild individuals in a mangrove forest of the Labuk Bay Proboscis Monkey Sanctuary fed on 17 plant species, supplemented by baked pancakes and cucumbers [34]. The extremely low dietary diversity in wild proboscis monkeys in a mangrove forest may be related to the absence of *L. nasalidis* and other *Lactobacillaceae* species. The intraspecific variation in the fore/hindgut microbial diversity has been demonstrated to decrease with changes in lifestyle from the wild to captivity and with a decrease in dietary diversity in the different habitats [19,35].

However, in spite of the low dietary diversity of the captive proboscis monkeys in a Malaysian zoo (only six plant species were consumed [19]), the 16S rRNA sequences of *L. nasalidis* and many other *Lactobacillaceae* species were detected in their samples (Table 1). In domestic pigs (*Sus scrofa domesticus*), domestication and a modern feeding system characterized by consuming high starchy food, may have induced the predominance of *Lactobacilli* over *Bifidobacterium* spp., which are predominantly lactate-producing bacteria in the wild boar (*Sus scrofa scrofa*) [36,37]. Furthermore, it was suggested that the amount of *Lactobacillaceae* species would be an indicator of reliance on anthropogenic food in Japanese macaques [38]. Therefore, the presence of *L. nasalidis* and many *Lactobacillus* species in a captive proboscis monkey might be caused and maintained by their humanized diet and/or close contact with humans in captivity, though it is required to consider other possibilities.

### 3.3. Comparison of Phenotype Characteristics between Captive and Wild Isolates

Of the 49 carbohydrates examined using the API 50CHL test, SR01 and SR02 strains metabolized 14 and 16 carbohydrates, respectively (Table S1). Of the 19 enzymes examined for their activity using APY ZYM, 9 were positive or weakly active in the SR01 strain and 8 in the SR02 strain (Table S2). Overall, these phenotypes were similar to those of the type strain YZ01^T^ (Table 2, Table S1 and Table S2) and were shown to be different from the closely related *L. delbrüeckii* subsp. *indicus* and *L. equicursoris*. 

For example, acid production from trehalose was observed in all seven strains of *L. nasalidis* irrespective of the host’s nature, but not in *L. delbrüeckii* subsp. *indicus* and *L. equicursoris*. Interestingly, the manner of trehalose degradation is important for proboscis monkeys. Trehalose is formed from two glucose molecules contained in plants and fungi and is the main blood sugar in insects. The trehalase gene, which encodes an enzyme capable of digesting trehalose, was intact in most primate species and the horse, but those of the two colobine monkeys (proboscis monkeys and golden snub-nosed monkeys) were disrupted by a shared mutation [39]. Therefore, it is suggested that proboscis monkeys are not capable of producing trehalase. Since trehalose was degraded by all seven strains of *L. nasalidis*, the latter bacterium can support the digestion of trehalose in proboscis monkeys.

Additionally, all seven strains of *L. nasalidis* fermented amygdalin, arbutin, salicin, cellobiose, and maltose, but not in *L. delbrüeckii* subsp. *indicus*. Among them, amygdalin, arbutin, and salicin are the typical secondary metabolites working as defense mechanisms for herbivores, especially amygdalin, which is toxic as a cyanogenic glycoside [40]. It has been suggested that colobine monkeys have a detoxification ability enhanced by the bacteria in their forestomachs; although, the details pertaining to these mechanisms are unknown [41]. Our current study successfully demonstrated that *L. nasalidis* degrades glycosides such as amygdalin and helps the proboscis monkey survive on plants rich in secondary metabolites. This type of symbiotic relationship has been observed in the Japanese rock ptarmigan (*Lagopus muta japonica*), a herbivore bird species that relies on alpine plants rich in secondary metabolites [42,43]. The relationship can be found in many wild herbivores, especially in folivores and frugivores.

On the other hand, some phenotypic differences were demonstrated between the presently isolated strains and the previously isolated typed strain YZ01^T^. Raffinose, which is a typical undigestible sugar, was weakly degraded by two strains (SR01 and SR02) of *L. nasalidis* isolated from the wild individuals. However, those from a captive individual did not degrade raffinose. Additionally, galactose was only degraded by SR02 (Table 2). Due to the more diverse diet of the wild proboscis monkeys, compared to that of captive individuals [33,44], the two strains isolated from the wild individuals degraded a wider variety of sugars than the strain YZ01^T^ isolated from a captive individual in Japan. Indeed, the wild proboscis monkey in the riverine forest where this study was conducted had a diet consisting of various proportions of leaves, fruits, and flowers including 188 plant species (127 genera and 55 families [33]). On the other hand, a captive individual in a Japanese zoo was fed a commercial pellet, supplemented with 16 plant species (16 genera and 12 families), including fruits, vegetables, beans, and seasonally available fresh leaves [44]. A similar tendency was observed in the gorilla (*Gorilla gorilla*), specifically *Limosilactobacillus gorillae* (formerly *Lactobacillus gorrilae*); strains from wild gorillas utilize a variety of different sugars as compared to those hosted by the captive gorillas [12].

Differences in NaCl tolerance were distinct between strains from a wild individual (SR01 and SR02) and strains from a captive individual (YZ01^T^, YZ02, and YZ05); SR01 and SR02 showed lower NaCl tolerance compared to the strainsYZ01^T^, YZ02, and YZ05 (Table 2). The same difference was observed in the gorilla-specific *Limosilactobacillus gorillae*. The differences in the sodium intake could be attributed to variations in the salt tolerance of the GIT bacteria [12]. The sodium levels in leaves consumed by proboscis monkeys inhabiting a riverine forest and a peat swamp forest are very low (10.1 ~ 27.4 mg/kg) [45]. On the other hand, the sodium content of diets consumed by individuals in the Singapore Zoo was reported to average 50.3 mg/kg [46], suggesting that *L. nasalidis* strains from captive individuals may generally be exposed to higher sodium intake than those from the wild monkeys. In addition, despite the failed bacterial isolation procedure, the 16S rRNA data in the previous study by Hayakawa et al. [18] revealed that *L. nasalidis* was present in a sample collected from a semi-wild provisioned individual living in a mangrove forest, where their food diets have been reported to contain an average of 1946 mg/kg [47], far higher than the sodium content in other forests and zoos. Hence, *L. nasalidis* may be highly tolerant to sodium, signifying that intraspecific variations in tolerance may emerge in response to the sodium content of the diet ingested by proboscis monkeys.

The function of the microbiome in the foregut of colobines has been discussed based on predictions using high-throughput sequencing of the 16S ribosomal RNA gene [48,49]. These discussions have often been conducted without validation and, in most cases, without physiological confirmation of the hypotheses derived, given that the utility of molecular microbiome studies for understanding the physiological functions of colobines remains limited when not coupled with physiological measurements and in vitro studies on the functions of the microorganisms involved [50]. Here, we clearly showed that phenotypic and physiological characteristics of proboscis monkey-specific LAB, *L. nasalidis*, varied among different hosts inhabiting diverse environments. 

## 4. Conclusions

In the present study, we successfully isolated *L. nasalidis* (which is unique to proboscis monkeys) from the freeze-dried forestomach contents of a wild individual that had been stored for over six years. The currently established method for the isolation of anaerobic bacteria from freeze-dried samples will be applicable to many already-existent precious samples.

Captive feeding environments modify considerably the GIT microbiome composition and induce the modification of the physiological functionalities of the same bacterial species. In the future, before the re-introduction of captive-bred proboscis monkeys to wild environments, acclimatization to wild food types is imperative so as to promote the proper development of their GIT microbiomes.

## Figures and Tables

**Table 1:** *Lactobacillaceae* species in the 16S metagenomic DNA library.

OUT_ID	Related Species	Similarity to Type Strain	Wild		Wild		Semi-Wild		Captive	
			Riverine Forest		Mangrove Forest		Mangrove Forest		Malaysian Zoo	
			Number ^a^	Proportion ^b^	Number ^a^	Proportion ^b^	Number ^a^	Proportion ^b^	Number ^a^	Proportion ^b^
OUT_391	*Lactobacillus nasalidis*	100.0	8	100.0	0	-	277	89.9	148	12.7
OUT_1195	*Lactobacillus delbrueckii subsp. lactis*	97.8	0	0.0	0	-	27	8.8	0	0.0
OUT_3049	*Lactobacillus equicursoris*	97.9	0	0.0	0	-	0	0.0	217	18.6
OUT_2284	*Lactobacillus plantarum*	100.0	0	0.0	0	-	1	0.3	0	0.0
OUT_1621	*Lactobacillus coleohominis*	97.6	0	0.0	0	-	0	0.0	6	0.5
OUT_223	*Lactobacillus mucosae*	95.2	0	0.0	0	-	0	0.0	575	49.4
OUT_3105	*Lactobacillus mucosae*	99.7	0	0.0	0	-	3	1.0	164	14.1
OUT_1269	*Lactobacillus mucosae*	95.7	0	0.0	0	-	0	0.0	35	3.0
OUT_2669	*Lactobacillus reuteri*	100.0	0	0.0	0	-	0	0.0	19	1.6
OUT_1535	*Lactobacillus agilis*	99.4	0	0.0	0	-	0	0.0	1	0.1
Subtotal reads for Lactobacillaceae			8		0		308		1165	
Total reads ^c^			40,809		40,809		40,809		40,809	

This table was constructed based on “Supporting Information Dataset S3” in Hayakawa et al. [19]. ^a^ Numbers of reads. ^b^ Proportion (%) to subtotal reads for *Lactobacillaceae* species. ^c^ Numbers of total reads in each environment were set to a minimum read size, 40,809, due to different sample sizes and read numbers.

**Table 2:** Phenotypic characteristics of strains of *Lactobacillus nasalidis* and related species.

	*L. nasalidis*							*L. delbrueckii* subsp. *indicus*	*L. equicursoris*
Isolated from	Wild Proboscis Monkey		Captive Proboscis Monkey					Indian Dairy Products	Thoroughbred Racehorse
	SR01	SR02	YZ01^T^ *	YZ02 *	YZ03 *	YZ04 *	YZ05 *	JCM 15610^T^ *	JCM 14600^T^ *
Acid production from (API 50 CHL)									
D-Galactose	−	+	−	−	−	−	−	−	+
D-Mannose	−	+	+	+	+	+	+	+	+
Amygdalin	+	+	+	+	+	+	+	−	W
Arbutin	+	+	+	+	+	+	+	−	+
Esculin ferric citrate	+	W	+	+	+	+	+	+	+
Salicin	+	+	+	+	+	+	+	−	+
D-Cellobiose	+	+	+	+	+	+	+	−	+
D-Maltose	+	+	+	+	+	+	+	−	+
D-Lactose	+	+	+	+	W	−	W	+	+
D-Melibiose	−	−	−	−	−	−	−	−	W
D-Trehalose	+	+	+	+	+	+	+	−	−
D-Raffinose	W	W	−	−	−	−	−	−	W
Starch	−	−	−	−	−	−	−	−	W
Gentiobiose	+	+	+	+	W	+	W	−	+
API ZYM results:									
Phosphate alkaline	−	−	−	−	−	W	W	−	−
Esterase (C 4)	W	−	W	W	W	W	W	W	W
Esterase lipase (C 8)	−	−	W	W	W	W	−	W	W
Lipase (C 14)	−	−	−	−	−	W	−	−	−
Leucine aminopeptidase	+	+	W	+	W	+	+	+	+
Valine aminopeptidase	−	−	−	W	W	W	W	W	−
Cystine aminopeptidase	W	W	−	W	−	W	−	W	W
Trypsin	−	−	−	−	−	−	−	−	W
Chymotrypsin	W	W	−	−	−	W	−	W	W
Phosphatase acid	W	W	+	+	+	+	+	+	W
Naphthol-AS-BI-phosphohydrolase	+	W	+	+	+	+	+	+	+
α-Galactosidase	−	−	−	−	−	−	−	W	+
β-Galactosidase	+	+	+	+	W	W	W	+	+
α-Glucosidase	W	W	W	W	W	W	W	−	+
α-Glucosidase	+	+	W	+	+	+	+	W	W
Growth at:									
15 °C	W	−	−	−	W	W	W	W	W
14 °C	+	+	+	+	+	+	+	+	+
Growth with NaCl (*w*/*v*)									
4.0%	+	+	+	+	+	+	+	+	−
5.0%	−	−	+	+	−	−	+	−	−
6.0%	−	−	−	−	−	−	−	−	−

* Data from Suzuki-Hashido et al. [14]. +, Positive; –, negative; w, weakly positive.

## Data Availability

Data are available from the corresponding author.

## Supplementary Materials

The following supporting information can be downloaded at: https://www.mdpi.com/article/10.3390/microorganisms11061458/s1, Table S1. Degradation of carbohydrates by *Lactobacillus nasalidis* and related species. Table S2. Enzyme activity *Lactobacillus nasalidis* and related species.
