# Supplementary material for: Isolation of Bacteria from Freeze-Dried Samples and the Functional Characterization of Species-Specific Lactic Acid Bacteria with a Comparison of Wild and Captive Proboscis Monkeys
Source: Microorganisms. 2023 May 31;11(6):1458. doi: 10.3390/microorganisms11061458 (PMC10305534; doi:10.3390/microorganisms11061458)
Supplement: Supplementary file 1 [file microorganisms-11-01458-s001.zip › microorganisms-2377005-supplementary.pdf]

Table S1. Degradation of carbohydrates by *Lactobacillus nasalidis* and related species.

	L. nasalidis							*L.delbrueckii* subsp. *indicus*	*L.equicursoris*
isolated from	wild proboscis monkey		captive proboscis monkey					Indian dairy products	thoroughbred racehorse
	SR01	SR02	YZ01[T *]	YZ02[*]	YZ03[*]	YZ04[*]	YZ05[*]	JCM 15610[T *]	JCM 14600[T *]
0 Control	-	-	-	-	-	-	-	-	-
1 Glycerol	-	-	-	-	-	-	-	-	-
2 Erythritol	-	-	-	-	-	-	-	-	-
3 D-Arabinose	-	-	-	-	-	-	-	-	-
4 L-Arabinose	-	-	-	-	-	-	-	-	-
5 D-Ribose	-	-	-	-	-	-	-	-	-
6 D-Xylose	-	-	-	-	-	-	-	-	-
7 L-Xylose	-	-	-	-	-	-	-	-	-
8 D-Adonitol	-	-	-	-	-	-	-	-	-
9 Methyl-β D−Xylopyranoside	-	-	-	-	-	-	-	-	-
10 D-Galactose	-	+	-	-	-	-	-	-	+
11 D-Glucose	+	+	+	+	+	+	+	+	+
12 D-Fructose	+	+	+	+	+	+	+	+	+
13 D-Mannose	-	+	+	+	+	+	+	+	+
14 L-Sorbose	-	-	-	-	-	-	-	-	-
15 L-Rhamnose	-	-	-	-	-	-	-	-	-
16 Dulcitol	-	-	-	-	-	-	-	-	-
17 Inositol	-	-	-	-	-	-	-	-	-
18 D-Mannitol	-	-	-	-	-	-	-	-	-
19 D-Sorbitol	-	-	-	-	-	-	-	-	-
20 Methyl-α D−Mannopyranoside	-	-	-	-	-	-	-	-	-
21 Methyl-α D−Glucopyranoside	-	-	-	-	-	-	-	-	-
22 N-Acetyl Glucosamine	+	+	+	+	+	+	+	+	+
23 Amygdalin	+	+	+	+	+	+	+	-	w
24 Arbutin	+	+	+	+	+	+	+	-	+
25 Esculin ferric citrate	+	w	+	+	+	+	+	+	+
26 Salicin	+	+	+	+	+	+	+	-	+
27 D-Cellobiose	+	+	+	+	+	+	+	-	+
28 D-Maltose	+	+	+	+	+	+	+	-	+
29 D-Lactose	+	+	+	+	w	-	w	+	+
30 D-Melibiose	-	-	-	-	-	-	-	-	w
31 D-Sucrose	+	+	+	+	+	+	+	+	+
32 D-Trehalose	+	+	+	+	+	+	+	-	-
33 Inulin	-	-	-	-	-	-	-	-	-
34 D-Melezitose	-	-	-	-	-	-	-	-	-
35 D-Raffinose	w	w	-	-	-	-	-	-	w
36 Starch	-	-	-	-	-	-	-	-	w
37 Glycogen	-	-	-	-	-	-	-	-	-
38 Xylitol	-	-	-	-	-	-	-	-	-
39 Gentiobiose	+	+	+	+	w	+	w	-	+
40 D-Turanose	-	-	-	-	-	-	-	-	-
41 D-Lyxose	-	-	-	-	-	-	-	-	-
42 D-Tagatose	-	-	-	-	-	-	-	-	-
43 D-Fucose	-	-	-	-	-	-	-	-	-
44 L-Fucose	-	-	-	-	-	-	-	-	-
45 D Arabitol	-	-	-	-	-	-	-	-	-
46 L Arabitol	-	-	-	-	-	-	-	-	-
47 Gluconate	-	-	-	-	-	-	-	-	-
48 2-Keto Gluconate	-	-	-	-	-	-	-	-	-
49 5-Keto Gluconate	-	-	-	-	-	-	-	-	-
+	13	14	14	14	12	13	12	7	13
w	1	2	0	0	2	0	2	0	4
-	36	34	36	36	36	37	36	43	33

* Data from Suzuki-Hashido et al. [14]. +, Positive; –, negative; w, weakly positive.

Table S2. Enzyme activity *Lactobacillus nasalidis* and related species.

	L. nasalidis							*L.delbrueckii* subsp. *indicus*	*L.equicursoris*
isolated from	wild proboscis monkey		captive proboscis monkey					Indian dairy products	thoroughbred racehorse
	SR01	SR02	YZ01^{T*}	YZ02*	YZ03*	YZ04*	YZ05*	JCM 15610^{T*}	JCM 14600^{T*}
1 Control	-	-	-	-	-	-	-	-	-
2 Phosphatase alkaline	-	-	-	-	-	w	w	-	-
3 Esterase (C 4)	w	-	w	w	w	w	w	w	w
4 Esterase lipase (C 8)	-	-	w	w	w	w	-	w	w
5 Lipase (C 14)	-	-	-	-	-	w	-	-	-
6 Leucine aminopeptidase	+	+	w	+	w	+	+	+	+
7 Valine aminopeptidase	-	-	-	w	w	w	w	w	-
8 Cystine aminopeptidase	w	w	-	w	-	w	-	w	w
9 Trypsin	-	-	-	-	-	-	-	-	w
10 Chymotrypsin	w	w	-	-	-	w	-	w	w
11 Phosphatase acid	w	w	+	+	+	+	+	+	w
12 Naphthol-AS-BI-phosphohydrolase	+	w	+	+	+	+	+	+	+
13 α-Galactosidase	-	-	-	-	-	-	-	w	+
14 β-Galactosidase	+	+	+	+	w	w	w	+	+
15 β-Glucuronidase	-	-	-	-	-	-	-	-	-
16 α-Glucosidase	w	w	w	w	w	w	w	-	+
17 β-Glucosidase	+	+	w	+	+	+	+	w	w
18 β-Glucosaminidase	-	-	-	-	-	-	-	-	-
19 α-Mannosidase	-	-	-	-	-	-	-	-	-
20 α-Fucosidase	-	-	-	-	-	-	-	-	-
+	4	3	3	5	3	4	4	4	5
w	5	5	5	5	6	9	5	7	7
-	11	12	12	10	11	7	11	9	8

* Data from Suzuki-Hashido et al. [14]. +, Positive; –, negative; w, weakly positive.
